# Post Cardiopulmonary Bypass Changes in Liver Function: Comment on "J Cardiovasc Thorac Res 2015;7:49-54"

**DOI:** 10.15171/jcvtr.2015.29

**Published:** 2015

**Authors:** Mehrdad Asgeri

**Affiliations:** Department of Medicine, Gastroenterology Section, Strong Memorial Hospital, University of Rochester, Rochester, NY, USA

This study examines abnormal LFT (mainly bilirubin) on postoperative day three as a predictor of open cardiac surgery mortality.^1^ Preoperative hyperbilirubinemia, high right atrial pressure, number of heart valves replaced and high blood transfusion requirements are risk factors predicting postoperative hyperbilirubinemia in 81% of all patients.^2^ This study strictly excluded high-risk conditions to cleverly evaluate the isolated and subtle liver injuries occurring post-cardiopulmonary bypass (CPB). As a result, 200 out of 400 patients originally enrolled were excluded which can lead to not sampling naturally distributed population. Most studies defined hyperbilirubinemia as total bilirubin >2 mg/dL for preoperative and >3 for postoperative whereas cut off was 1.5 mg/dl in this study.^2,3^ The effects of diabetes (which causes direct hyperbilirubinemia) and BMI status on liver function are unknown.^4^ The study examined excreting function of liver only (bilirubin), but neither synthetic (INR, albumin) nor metabolic (mono-ethyl glycine xylidide) markers of liver function were not measured.^5^ All CPB operations were on pump, with none-pulsatile perfusion, average amount of blood transfusion (no autologous blood) with systemic hypothermia of 28°C.

Results showed minimal increase in TB by 0.2 mg/dl (mainly direct bilirubin), AST and ALP on postoperative day three. These changes mostly occurred in patients with preoperative hyperbilirubinemia, which was consistent with other studies.^2^ Transient benign increase in LFT can occur post CPB. Hyperbilirubinemia could be related to undetected RV failure, undetected preoperative hyperbilirubinemia and prolonged intraoperative hypotension (multiple etiologies possible). The study excluded patients with overt preoperative congestive heart failure, but did not measure intraoperative central filling pressures. Some patients had preoperative hyperbilirubinemia of unknown etiology. Prolonged intraoperative hypotension could occur due to multiple reasons including perioperative myocardial infarction, hypovolemia, low cardiac output, lower pump flow of non-pulsatile CPB and prolonged CPB time.

Hyperbilirubinemia of 2.5 and >3.7 on post-op day two and seven, respectively, were predictors of the mortality in other studies.^2,6^ These studies included high-risk patients including cardiac valve replacement. Of four mortalities reported, two were due to liver failure with total bilirubin >24 on seventh postoperative day and two others died of multi-organ failure. Among the patients with cardiac transplant, the mortality was 25% in patients with postoperative total bilirubin > 2.8 mg/dL which was much greater than 4.3% in patients with a total bilirubin between 1.4 and 2.8 mg/dL. Additionally, two year post-CPB survival decreased by 1.7 fold vs. 3.8 fold with postoperative total bilirubin of 1.4-2.8 vs. >2.8, respectively.^7^

As the mortality data were not included, it was difficult to conclude whether hyperbilirubinemia on the third postoperative day could serve as the single strong predictor of jaundice-related mortality. A relatively lower incidence was reported for jaundice and that was probably probably due to healthy patient selection. Yet, the reported mortality due to liver failure among the jaundiced patients was significantly higher than that reported by others. Higher transfusion requirement (>6 units), possibility of infection along with the above mentioned factors could be contributing. Pump time was 100 minutes. Pump time >70 min associated with post-op hyperbilirubinemia; >120 minutes associated with high mortality.^5,8^ Hypothermia benefits were lost due to intraoperative hypotension and longer duration of anesthesia. Hyperbilirubinemia on post op day three should be aggressively investigated and treated. Anatomically suitable patients may benefit from off-pump cardiac surgery and coronary grafting.^9^

## Ethical issues

Not applicable.

## Competing interests

Author declares no conflict of interest in this study.
